# Targeting MEK in vemurafenib-resistant hairy cell leukemia

**DOI:** 10.1038/s41375-018-0270-2

**Published:** 2018-10-19

**Authors:** Rebecca Caeser, Grace Collord, Wen-Qing Yao, Zi Chen, George S. Vassiliou, Philip A. Beer, Ming-Qing Du, Mike A. Scott, George A. Follows, Daniel J. Hodson

**Affiliations:** 1 Department of Haematology, University of Cambridge, Cambridge, UK; 2 Wellcome-MRC Cambridge Stem Cell Institute, Cambridge, UK; 3 Wellcome Sanger Institute, Hinxton, UK; 4 Department of Paediatrics, University of Cambridge, Cambridge, UK; 5 Division of Molecular Histopathology, University of Cambridge, Cambridge, UK; 6 Haematopathology & Oncology Diagnostic Service, Cambridge University Hospitals, Cambridge, UK; 7 Department of Haematology, Cambridge University Hospitals, Cambridge, UK

**Keywords:** Medical research, Translational research

Hairy cell leukemia (HCL) is a chronic, incurable B cell malignancy that presents with splenomegaly, bone marrow infiltration, and cytopenias [1]. Long remissions are typically achieved with purine analogs such as cladribine, but most cases will relapse and require further therapy. The discovery of the *BRAF* V600E mutation in almost all cases of HCL [2] has led to the widespread adoption of the BRAF inhibitor vemurafenib for treatment of patients relapsing after cladribine [3–5]. Impressive responses are reported; however, relapse is inevitable [5, 6] and hematologists are now faced with a growing number of patients with vemurafenib-resistant HCL. The optimal management of these patients remains unclear.

The molecular basis of vemurafenib resistance has been extensively investigated in recent years in patients with *BRAF* mutant solid organ malignancies such as melanoma and colorectal cancer [7]. Resistance to vemurafenib in melanoma frequently results from reactivation of ERK pathway signaling by a variety of genetic mechanisms that include activating mutations of *NRAS* or *KRAS*, amplification of mutant *BRAF*, aberrant splicing of *BRAF*, and activating mutation of *MAP2K1*, which encodes the MEK1 protein [7, 8]. ERK-independent mechanisms are less frequent and include activation of PI3K signaling [7]. The predominance of genetic mechanisms converging on ERK reactivation has led to the successful use of dual MEK/BRAF inhibition in melanoma [9]. In colorectal cancer however, different mechanisms apply; here primary resistance usually results from reduced feedback inhibition upon upstream receptor tyrosine kinase activity leading to restoration of ERK activity [10]. In this scenario, combined BRAF and MEK inhibition has not proved effective [11].

In contrast to our comprehensive understanding in solid organ cancer, very little is known about the mechanistic basis of vemurafenib resistance in HCL. Given the diversity of resistance mechanisms established in other cancers, it is unclear which, if any, of these might predominate in HCL. Two acquired subclonal, activating *KRAS* mutations were previously reported in a single patient with vemurafenib resistance [5]. Deletions of *NF1* and *NF2* have been proposed as an alternative mechanism in another case of primary resistance [12]. The use of MEK inhibition has been suggested as a logical therapeutic strategy in patients who have reactivated ERK signaling. However, the use of MEK inhibition has never previously been reported in a patient with HCL and at present there is no consensus on the optimal management of patients relapsing on vemurafenib.

A 74-year-old patient with HCL had been treated at our institution with splenectomy, cladribine, and pentostatin. We previously reported his initial response to vemurafenib at a dose of 240 mg twice daily [4]. This dose was lower than used in the initial phase II trial [5], but has since been shown in several reports to be an effective dosing strategy for HCL [3, 13, 14]. Vemurafenib was initially stopped after 58 days; however, this was associated with rapid return of marrow infiltration and thrombocytopenia. Vemurafenib was restarted at the same dose and cytopenias rapidly resolved. Continuous low-dose vemurafenib continued to sustain his remission for over 3 years, attesting to the efficacy of this dosing schedule. However, 38 months after restarting vemurafenib, his blood indices deteriorated, and he required platelet transfusion (Fig. 1a). Bone marrow trephine biopsy confirmed relapse of HCL. A trial of rituximab with continued vemurafenib led to transient recovery of hematological indices. However, bone marrow infiltration did not improve over the next 4 months, and the patient became anemic, thrombocytopenic, and required further platelet transfusion. A second trial of two doses of rituximab produced a minimal improvement of platelet count to 30 × 10^9^/l. The patient became systemically unwell with B symptoms. Bone marrow trephine biopsy confirmed 99% infiltration with HCL.

**Fig. 1:**
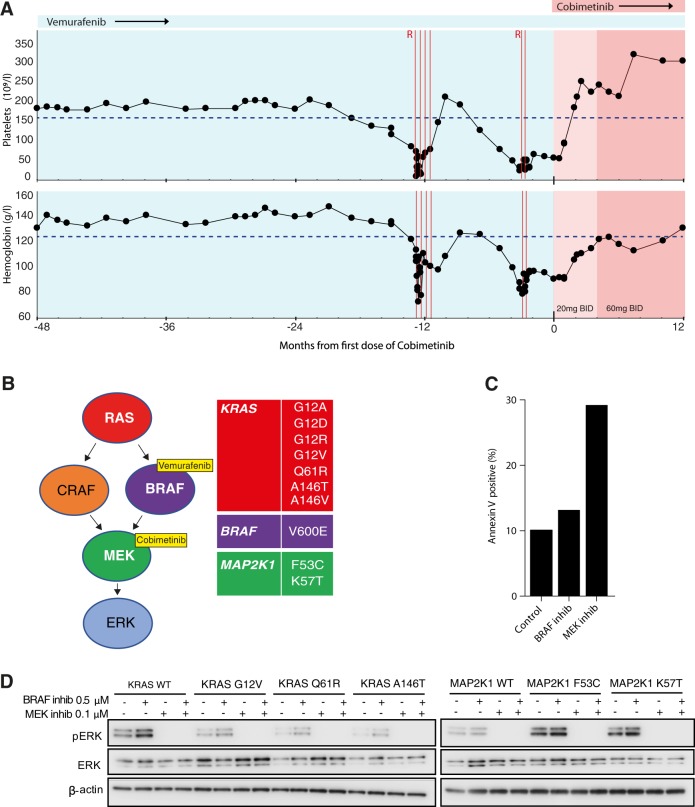
**a** The patient’s peripheral blood indices are shown over time relative to the first dose of the MEK inhibitor cobimetinib. Vertical red lines indicate the timing of rituximab dosing. Blue shading indicates vemurafenib monotherapy 240 mg twice daily (vem mono). Pale pink shading indicates vemurafenib with cobimetinib 20 mg daily (cobi-20). Darker pink indicates vemurafenib with cobimetinib 60 mg daily (21/28 days) (cobi-60). The lower limits of normal reference values are indicated by horizontal dashed lines. **b** Schematic of the MEK-ERK signaling pathway with mutations identified in purified tumor cells after emergence of resistance to vemurafenib. **c** Annexin V staining was used to quantify the induction of apoptosis in tumor cells purified from the patient and incubated for 48 h ex vivo with inhibitors of BRAF (vemurafenib) or MEK (trametinib). Apoptosis is induced by MEK inhibition but not by BRAF inhibition. **d** Immunoblots of a lymphoma cell line transduced with the indicated *KRAS* or *MAP2K1* constructs and incubated with inhibitors of BRAF or MEK. Complete suppression of ERK activity is seen with MEK inhibition but not with BRAF inhibition

To elucidate the mechanism of his resistance we performed whole-genome and deep-targeted sequencing of 292 genes (Supplementary Table 1) of DNA from purified tumor cells collected prior to starting vemurafenib and again at relapse. Samples were used with informed written patient consent in accordance with the Declaration of Helsinki and appropriate institutional ethical approvals. Sequencing studies revealed the presence of the known *BRAF* V600E mutation and chromosome 7q deletion. Remarkably, we also identified seven distinct activating mutations in *KRAS* and two mutations in *MAP2K1* (encoding MEK1) (Fig. 1b and Supplementary Table 2). These were detectable at relapse but were not detectable prior to vemurafenib exposure. Allele frequencies were consistent with the parallel, convergent evolution of multiple clones. Deep-targeted amplicon sequencing at multiple time points showed how *KRAS* mutations developed early, initially with codon 146 mutations, followed by emergence of the more classical activating mutations of codons 12 and 61 [15]. *MAP2K1* mutations appeared later with *MAP2K1* p.K57T expanding to become the dominant clone (Fig. 2c and Supplementary Table 2). The convergent nature of these mutations strongly implicated reactivation of MEK-ERK signaling as the likely mechanism of resistance. Indeed, immunohistochemistry confirmed strong pERK activity in all tumor cells (Fig. 2a). We looked for other mechanisms of resistance reported in melanoma. Specifically, we found no genetic or protein evidence of either increased pAKT activity or altered BRAF splicing (Supplementary Figure 1A & B).

**Fig. 2:**
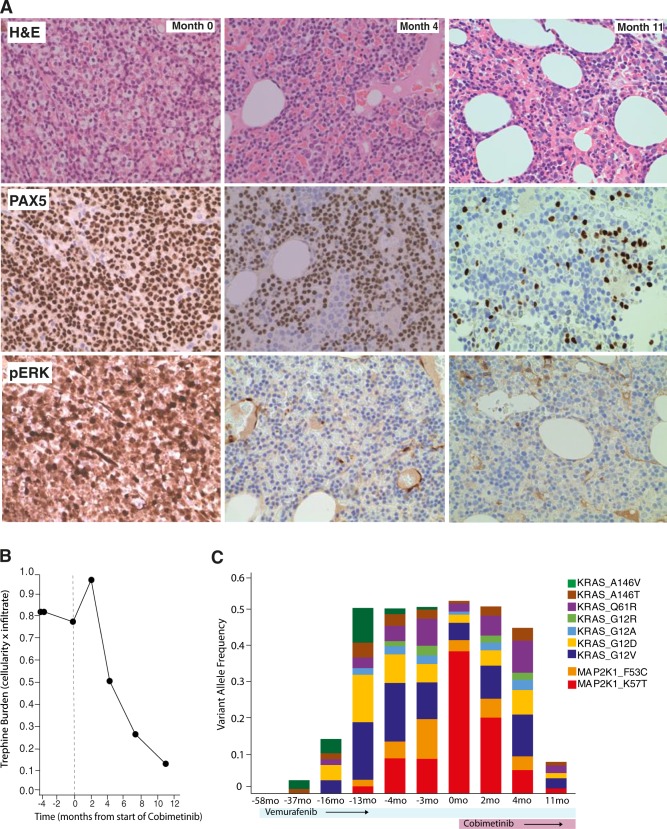
**a** Bone marrow trephine biopsies stained with H&E (top) or PAX5 antibody (middle) or pERK (lower) taken at the indicated time points relative to start of cobimetinib. **b** Leukemic burden prior to and after starting cobimetinib therapy was calculated as the product of bone marrow trephine cellularity and leukemic cell infiltrate. **c** Mutant allele frequency for the indicated *KRAS* and *MAP2K1* mutations quantified by targeted amplicon sequencing at multiple time point relative to treatment

We concluded that reactivation of MEK/ERK activity was the most likely driver of relapse and hypothesized that MEK inhibition might be a successful therapeutic strategy. Expression of the KRAS and MAP2K1 mutants in a lymphoid cell line showed that while each mutation was able to activate ERK in the presence of vemurafenib, all mutations remained sensitive to MEK inhibition (Fig. 1d). Exposure of the patient’s purified tumor cells to vemurafenib ex vivo failed to completely suppress ERK activity and did not induce apoptosis. In contrast, ERK activity was completely suppressed and apoptosis induced by exposure to a MEK inhibitor (Supplementary Figure 1C and Fig. 1c).

Based on our in vitro experiments, we treated the patient with the MEK inhibitor cobimetinib, initially at 20 mg daily combined with vemurafenib 240 mg twice daily. Remarkably, B symptoms resolved within 1 week, followed by rapid platelet count recovery. A bone marrow biopsy at 4 months showed complete suppression of ERK activity (Fig. 2a). However, HCL marrow infiltration persisted, and therefore cobimetinib dose was increased to 60 mg daily (taken 21 out of 28 days). The dose was well tolerated and was associated with further resolution of cytopenias, a substantial reduction in bone marrow cellularity and HCL infiltration, ongoing suppression of ERK activity and restoration of normal hematopoiesis (Fig. 2a, b). Ongoing treatment was also associated with suppression of mutant allele frequencies for *BRAF*, *KRAS*, and *MAP2K1* mutations (Fig. 2c). At 12 months, the patient remains well and asymptomatic with continued combination therapy.

The finding of nine activating mutations, all converging upon the activation of RAS/RAF/MEK/ERK signaling, underscores the centrality of this pathway in HCL and its reactivation as a potent mechanism of resistance to vemurafenib in this disease. This report provides a detailed analysis of the molecular basis for acquired vemurafenib resistance in HCL. It is the first reported use of a MEK inhibitor to treat vemurafenib-resistant HCL. It proposes a new therapeutic option for such patients and provides impetus for testing in a formal trial setting.

## Electronic supplementary material

Supplementary
