# Supplementary material for: Targeting MEK in vemurafenib-resistant hairy cell leukemia
Source: Leukemia. 2018 Oct 19;33(2):541–5. doi: 10.1038/s41375-018-0270-2 (PMC6365378; doi:10.1038/s41375-018-0270-2)
Supplement: Supplementary file 1 — Supplementary [file 41375_2018_270_MOESM1_ESM.pdf]

Targeting MEK in vemurafenib-resistant hairy cell leukemia.

Supplementary Information

Methods

1. Patient Samples

Samples were obtained with written informed consent for tumor banking and DNA sequencing. The study protocol was approved by the East of England Cambridge South Research Ethics Committee (approval reference number 07/MRE05/44). Mononuclear cells from peripheral blood or disaggregated bone marrow trephine were isolated using a density gradient medium (Lymphoprep, STEMCELL) following the manufacturer's instructions. CD19-positive B cells were then depleted using CD19 MicroBeads (MACS Miltenyl Biotec) and a MACS Separator.

2. Apoptosis assay

1 million CD19-positive HCL cells were treated with 1 µM vemurafenib (Selleckchem, S1267) or 100 nM trametinib (Selleckchem, S2673) for 48 hours. APC-Annexin V/Dead Cell Apoptosis Kit (Life Technologies) was used for the detection of apoptotic cells according to the manufacturer's instructions. Externalization of phosphatidylserine (Annexin V-APC Conjugate) and DNA content (DAPI, 4',6-diamidino-2-phenylindole) were measured using a Flow Cytometer and gating on all cells was used for further analysis.

3. Western blotting

Cell extracts were prepared as follows: 1 million cells were taken after 2 hours of drug treatment and lysed with LDS Buffer and 2-ME followed by loading the same amount per sample on NuPAGE (Invitrogen) 4-12% Bis-Tris gradient gels and were further transferred on PVDF membranes (Invitrogen). Primary antibodies (Phospho-p44/42 MAPK (Erk1/2) (Thr202/Tyr204), p44/42 MAPK (Erk1/2), Phospho-Akt (Ser473), Total Akt, Raf-B and β-Actin) were used with the HRP immunodetection system (Life Technologies/Millipore) to detect human proteins. Western blot antibodies were obtained from Cell Signalling Technology and Santa Cruz.

4. DNA extraction

Cells were resuspended in a final volume of 600 µl Lysis Buffer and 15 µl Proteinase K solution. After incubation at 65° for 15 min, genomic DNA was precipitated using isopropanol and resuspended in TE Buffer.

5. Plasmid Construction and viral transduction

To generate the KRAS and MEK1 plasmids, the CDS sequences for KRAS WT (NM_004985, NCBI) and MAP2K1 WT (NM_002755, NCBI) were designed as G-blocks and purchased from IDT. These were cloned into the XhoI/HindIII-digested pRCMV-TOP vector using Gibson Assembly.

KRAS mutations (G12V, Q61R, A146T) and MAP2K1 mutations (F53C, K57T) were generated from the pRCMV-TOP-KRAS/MAP2K1 WT plasmid. Mutation sequences were obtained from IDT as G-Bocks and cloned into the BamHI and HindIII-digested pRCMV-TOP-KRAS and MAP2K1 WT, respectively, using Gibson Assembly. All plasmids were verified by capillary sequencing. 293T cells (Clontech) were transfected with relevant KRAS/MAP2K1 and packaging plasmids using TransIT-293 (Mirus). Viral supernatant was collected at 48 hours, filtered (0.48micron) and spinoculated into BJAB cells maintained in RPMI with 10% FCS.

6. Sequencing

6.1 Whole genome sequencing and cRNA bait pulldown targeted sequencing

6.1.1 Library Preparation

For whole genome sequencing, short insert (500bp) genomic libraries were constructed, flowcells prepared and 150 base pair paired-end sequencing clusters generated on the Illumina HiSeq X platform according to Illumina no-PCR library protocols[1]. The average sequence coverage was 52.5X for tumor samples and 30.3X for matched normal sample.

For targeted sequencing we used a custom complementary RNA (cRNA) bait set (SureSelect, Agilent, UK, ELID # 0731661) to enrich for all coding exons of 292 genes implicated in haematological cancers (supplementary table 1). Short insert libraries (150bp) were prepared and sequenced on the Illumina HiSeq 2000 using 75 base paired-end sequencing as per Illumina protocol with 3 samples multiplexed per lane. Mean sequence coverage was 941.7X.

6.1.2 Sequencing data alignment

DNA sequencing reads were aligned to the GRCh 37d5 reference genome using the Burrows-Wheeler transform (BWA); BWA-MEM and BWA-aln were used for whole genome and targeted sequencing, respectively[2,3]. For targeted sequencing, PCR duplicates and reads mapping to regions outside target regions (merged exonic regions + 10bp either side of each exon) were excluded from analysis. Sequencing depth at each base was assessed using Bedtools coverage v2.24.0[4]. Point mutation variants were annotated using VAGrENT[5] according to ENSEMBL version 58.

6.1.3 Variant calling from whole genome sequencing data

6.1.3.1 Point mutations

Single base somatic substitutions were called using an in-house version of CaVEMan v1.11.2 (Cancer Variants through Expectation Maximization)[6]. CaVEMan compares sequencing reads from tumor and matched normal samples and uses a naïve Bayesian model and expectation-maximization approach to calculate the probability of a somatic variant at each base

(https://github.com/cancerit/CaVEMan). Post-processing filters required that the following criteria were met to call a somatic substitution:

1. At least a third of the reads calling the variant had a base quality of 25 or higher.
2. If coverage of the mutant allele was less than 8, at least one mutant allele was detected in the first 2/3 of the read.
3. Less than 5% of the mutant alleles with base quality ≥ 15 were found in the matched normal.
4. Not all mutant alleles reported in the second half of the read.
5. Mean mapping quality of the mutant allele reads was ≥ 21.
6. Mutation does not fall in a simple repeat or centromeric region.
7. Position does not fall within a germline insertion or deletion.
8. Variant is not reported by ≥ 3 reads in more than one percent of samples in a panel of approximately 400 unmatched normal samples.
9. A minimum 2 reads in each direction reporting the mutant allele.
10. At least 10-fold coverage at the mutant allele locus.
11. Minimum variant allele fraction 5%.
12. No insertion or deletion called within a read length (150bp) of the putative substitution.
13. No soft-clipped reads reporting the mutant allele.
14. Median BWA alignment score of the reads reporting the mutant allele ≥ 140.

Small insertions and deletions were sought using an in-house version of Pindel v2.2.2 [7] (https://github.com/cancerit/cgpPindel). Post-processing filters required that the following criteria were met for a variant to be called:

1. Minimum of 2 reads in each direction reporting the variant (read count based on the union of BWA and Pindel reads reporting the mutant allele) and minimum VAF 5%
2. Variant not present within an unmatched normal panel of approximately 400 samples.
3. No reads supporting the variant identified in the matched normal sample.
4. Minimum coverage >10.

All mutations meeting potential driver criteria were manually inspected in the Jbrowse genome viewer[8] prior to being discarded.

6.1.3.2 Rearrangements

Structural variants were called using a bespoke algorithm, BRASS v12.3.1 (https://github.com/cancerit/BRASS). Only breakpoints that could be validated by reconstruction at a base pair resolution are reported.

6.1.3.3 Copy number variants

The ascatNGS algorithm (v4.0.1)[9] as used to estimate tumor purity and ploidy and to construct copy number profiles.

6.1.4 Variant calling from deep targeted sequencing

Somatic single nucleotide variants (SNVs) were called using deepSNV, an algorithm developed for detecting subclonal mutations in deep sequencing experiments (https://github.com/gerstung-lab/deepSNV) [10]. Only reads with minimum nucleotide and mapping quality of 25 and 40, respectively, were considered. This algorithm models the error rate at individual loci using information from multiple unrelated samples. Allele counts at detected mutations were generated using an in-house script (https://github.com/cancerit/alleleCount) and manually inspected in the Jbrowse genome browser [8].

Small insertions and deletions (indels) were sought using an in-house version of Pindel as described in Methods 12.1.2 complemented with t the aforementioned deepSNV algorithm in order to increase sensitivity for indels present at low VAF. VAF correction was performed using an in-house script (https://github.com/cancerit/vafCorrect).

6.1.5 Curation of oncogenic variants

We considered variants as potential drivers or RAF inhibitor resistance events if they met any of the following criteria:

1. Variants in tumor suppressor genes were considered if they occurred within 3 amino acids of a recurrent hotspot mutation or were deleterious (nonsense, essential splice site or frameshift indel, disruptive rearrangement breakpoints or focal (<1 Mb) homozygous deletions).
2. Mutations in oncogenes were considered potential driver events if they were located at previously reported canonical hot spots (point mutations) or involved focal (<1Mb) amplification (>5 copies) of the intact gene.
3. Non-synonymous mutations in genes involved in the RAF pathway or previously implicated in RAF inhibitor resistance[11].

7. Targeted Amplicon Sequencing

The genomic sequences spanning the *KRAS*, *BRAF* and *MAP2K1* mutations identified by whole genome and deep targeted sequencing were amplified by PCR in duplicate (**Supplementary Table 3**), then routinely purified, barcoded and pooled for Illumina MiSeq sequencing[12]. The data analyses and variant calls were carried out as described previously[13], then curated by manual inspection of sequencing reads. The variants that appeared in both replicates and were above the assay sensitivity threshold (>0.7% VAF, defined by mean + 3SD of background noise from non-neoplastic samples) were retained.

8. Phospho-ERK Immunohistochemistry

Immunostaining for phospho-ERK was performed on fixed, decalcified trephine biopsy material using pERK antibody from Cell Signaling Technology (Rabbit D13.14.4E, #4370) at 1:400 dilution with antigen retrieval using HIER for 20 minutes and detected using Bond™ Polymer Refine Detection.

Data Availability

DNA sequencing data can be accessed at the European Genome-Phenome Archive, study accession numbers EGAD00001003923 and EGAD00001003924. All other relevant data are available from the corresponding author upon reasonable request.

Supplementary References

1. Kozarewa I, Ning Z, Quail MA, Sanders MJ, Berriman M, Turner DJ. Amplification-free Illumina sequencing-library preparation facilitates improved mapping and assembly of (G+C)-biased genomes. Nat Methods 2009;6:291-5.
2. Li H, Durbin R. Fast and accurate short read alignment with Burrows-Wheeler transform. Bioinformatics 2009;25:1754-60.
3. Li H, Durbin R. Fast and accurate long-read alignment with Burrows-Wheeler transform. Bioinformatics 2010;26:589-95.
4. Quinlan AR, Hall IM. BEDTools: a flexible suite of utilities for comparing genomic features. Bioinformatics 2010;26:841-2.
5. Menzies A, Teague JW, Butler AP, et al. VAGrENT: Variation Annotation Generator. Curr Protoc Bioinformatics 2015;52:15 8 1-1.
6. Jones D, Raine KM, Davies H, et al. cgpCaVEManWrapper: Simple Execution of CaVEMan in Order to Detect Somatic Single Nucleotide Variants in NGS Data. Curr Protoc Bioinformatics 2016;56:15 0 1- 0 8.
7. Raine KM, Hinton J, Butler AP, et al. cgpPindel: Identifying Somatically Acquired Insertion and Deletion Events from Paired End Sequencing. Curr Protoc Bioinformatics 2015;52:15 7 1-2.
8. Buels R, Yao E, Diesh CM, et al. JBrowse: a dynamic web platform for genome visualization and analysis. Genome Biol 2016;17:66.
9. Van Loo P, Nordgard SH, Lingjaerde OC, et al. Allele-specific copy number analysis of tumors. Proc Natl Acad Sci U S A 2010;107:16910-5.
10. Gerstung M, Beisel C, Rechsteiner M, et al. Reliable detection of subclonal single-nucleotide variants in tumour cell populations. Nat Commun 2012;3:811.
11. Holderfield M, Deuker MM, McCormick F, McMahon M. Targeting RAF kinases for cancer therapy: BRAF-mutated melanoma and beyond. Nat Rev Cancer 2014;14:455-67.
12. Wang M, Escudero-Ibarz L, Moody S, et al. Somatic Mutation Screening Using Archival Formalin-Fixed, Paraffin-Embedded Tissues by Fluidigm Multiplex PCR and Illumina Sequencing. J Mol Diagn 2015;17:521-32.
13. Cucco F, Clipson A, Kennedy H, et al. Mutation screening using formalin-fixed paraffin-embedded tissues: a stratified approach according to DNA quality. Lab Invest 2018.

Supplementary Figure 1

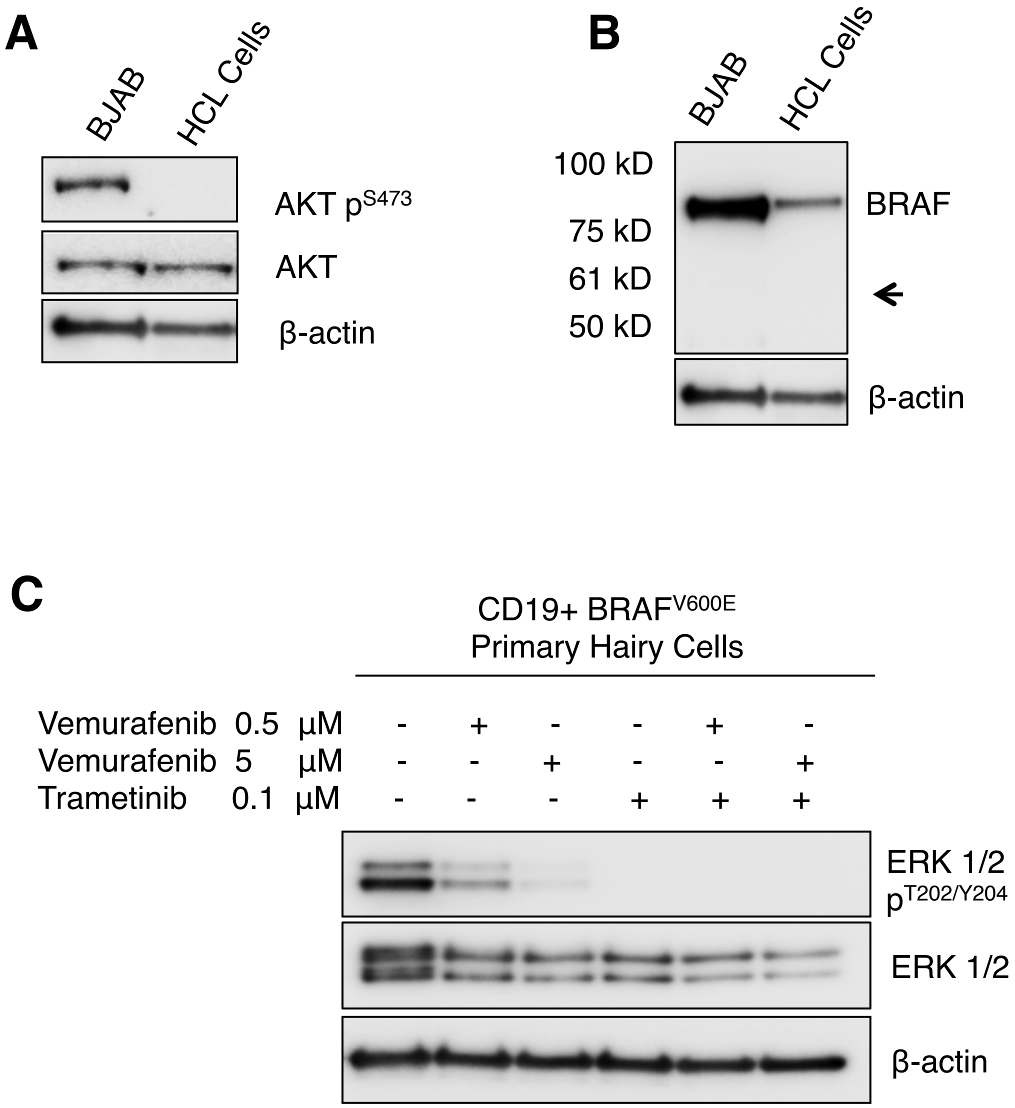

A. CD19 purified leukemic cells isolated from the peripheral blood of the patient at the time of his relapse show no activation of AKT signaling. The lymphoma cell line BJAB is included as a positive control.

B. CD19 purified leukemic cells isolated at the time of the patient's relapse show no evidence of aberrantly spliced BRAF. The lymphoma cell line BJAB is included as a control to confirm the position of WT BRAF. The expected position of an aberrantly spliced BRAF (61 kD) is indicated with black arrow.

C. CD19 positive leukemic cells isolated at the time of relapse were treated with the indicated concentrations of vemurafenib, the MEK inhibitor trametinib or the combination of both for 2h before immunoblotting for phospho and total ERK. Vemurafenib fails to completely suppress ERK activity even at high concentration, whereas complete suppression is seen with a MEK inhibitor.

Supplementary Table 1.

292 genes sequenced by deep targeted sequencing

ABL1	EGR1	KMT2E	REL
ACTG1	EGR2	KRAS	RELN
AKAP9	EP300	KSR2	RET
ALK	ERBB2	LCK	RHOA
APC	ERCC4	LTB	ROBO1
ARAF	ETNK1	LYN	ROS1
ARID1A	ETV1	MALT1	RPL10
ARID1B	ETV6	MAP2K1	RPL5
ARID2	EZH2	MAP2K4	RPN1
ASXL1	FAM46C	MAX	RPS15
ASXL2	FAS	MECOM	RPS2
ATM	FAT1	MED1	RUNX1
ATRX	FAT2	MEF2B	S1PR2
B2M	FAT3	MET	SAMHD1
BACH2	FAT4	MGA	SETBP1
BCL10	FBXO11	MLH1	SETD2
BCL11A	FBXW10	MPEG1	SETDB1
BCL2	FBXW7	MPL	SF1
BCL6	FGFR2	MSH2	SF3A1
BCL7A	FGFR3	MSH6	SF3B1
BCOR	FLT3	MTOR	SGK1
BCORL1	FNDC3A	MYB	SH2B3
BCR	FOXO1	MYC	SMARCA4
BIRC2	FYN	MYD88	SMARCB1
BIRC3	GATA1	NBEAL2	SMC1A
BLM	GATA2	NF1	SMC3
BLNK	GATA3	NF2	SNX7
BRAF	GCSAM	NFE2	SOCS1
BTG1	GMPS	NFKB1	SP140
BTG2	GNA13	NFKB2	SPEN
BTK	GNAS	NFKBIA	SPIB
CALR	H3F3A	NFKBIE	SPRY4
CARD11	H3F3B	NFKBIZ	SRSF2
CASP8	HIST1H1B	NOTCH1	STAG2
CBFB	HIST1H1C	NOTCH2	STAT3
CBL	HIST1H1D	NPM1	STAT5B
CCND1	HIST1H1E	NRAS	STAT6
CCND3	HIST1H2AC	NTRK1	STK11
CD19	HIST1H2AG	P2RY8	SUZ12
CD22	HIST1H2AM	PAX5	SYK
CD274	HIST1H2BC	PBRM1	TBL1XR1
CD58	HIST1H2BD	PDGFRA	TCF3
CD70	HIST1H2BG	PDGFRB	TET1
CD79A	HIST1H3B	PDK1	TET2
CD79B	HIST1H3G	PDS5B	TET3
CD83	HLA-A	PEG3	TGDS
CDK4	HLA-B	PHF6	THPO
CDKN1B	HLA-C	PIK3CA	THRAP3
CDKN2A	HRAS	PIK3CD	TLR2
CDKN2B	ID3	PIK3R1	TMEM30A
CDKN2C	IDH1	PIM1	TNF
CEBPA	IDH2	PLCG1	TNFAIP2
CHD2	IKBKG	PLCG2	TNFAIP3
CHD4	IKZF1	PMS2	TNFRSF14
CHEK2	IKZF3	POT1	TNKS
CHUK	IL2RG	POU2AF1	TP53
CIITA	IL7R	POU2F2	TP73
CREBBP	INTS12	PPM1D	TRAF2
CSF2RB	IRF1	PRDM1	TRAF3
CSF3R	IRF4	PRKCB	TYK2
CTCF	IRF8	PRPF40B	U2AF1
CTNNB1	ITPKB	PTCH1	U2AF2
CUX1	JAK1	PTEN	USP29
CXCR4	JAK2	PTPN1	USP6
CYLD	JAK3	PTPN11	WHSC1
DDX3X	KDM4C	PTPN6	WT1
DIS3	KDM6A	PTPRC	XBP1
DNMT3A	KDR	PTPRD	XPO1
DNMT3B	KIT	RAD21	ZFP36L1
DTX1	KLF2	RAD51	ZRSR2
EBF1	KLHL6	RAG2	
ECT2L	KMT2A	RASA2	
EED	KMT2C	RB1	
EGFR	KMT2D	RCOR1	

Supplementary Table 2. Variant Allele Fequency for each mutation at time-points relative to start of cobimetinib

Time-point (months)	Treatment	Method	Seq ID	DNA Source	BRAF_c.T1799A_p. V600E	MAP2K1_c.T158G_p. F53C	MAP2K1_c.A170C_p. K57T	KRAS_c.G35T_p. G12V	KRAS_c.G35A_p. G12D	KRAS_c.G35C_p. G12A	KRAS_c.G34C_p. G12R	KRAS_c.A182G_p. Q61R	KRAS_c.G436A_p. A146T	KRAS_c.C437T_p. A146V
**-58**	Vem Start	Amplicon	HCL01	CD19 PB	0.77									
**-58**	Vem Start	**WGS**	PD29747a	CD19 PB	0.77									
**-16**	Vem	Amplicon	HCL03	BM	0.17			0.04	0.04			0.02	0.02	0.04
**-16**	Vem	**Pulldown**	PD29747d	BM	0.17			0.03	0.04			0.00	0.02	0.03
**-13**	Vem	Amplicon	HCL04	CD19 PB	0.99	0.02	0.02	0.16	0.13	0.02		0.03	0.04	0.10
**-13**	Vem	**WGS**	PD29747c	CD19 PB	0.72								0.08	0.15
**-4**	Vem	Amplicon	HCL05	CD19 PB	0.93	0.05	0.10	0.16	0.08	0.02	0.01	0.04	0.03	0.01
**-3**	Vem	Amplicon	HCL07	BM	0.87	0.11	0.10	0.10	0.05	0.02	0.03	0.07	0.03	0.01
**-3**	Vem	**Pulldown**	PD29747e	BM	0.88	0.12	0.08	0.06	0.02	0.02	0.01	0.05	0.02	
**0**	Vem /Cobi-20 Start	Amplicon	HCL08	CD19 BM	0.95	0.03	0.39	0.05	0.02	0.01		0.02	0.01	
**2**	Vem /Cobi-20	Amplicon	HCL09	CD19 BM	0.89	0.05	0.21	0.09	0.04	0.02	0.02	0.06	0.03	
**4**	Vem /Cobi-60	Amplicon	HCL10	CD19 BM	0.68	0.04	0.07	0.12	0.07	0.03	0.02	0.09	0.04	
**11**	Vem /Cobi-60	Amplicon	HCL12	CD19 BM	0.10		0.01	0.03	0.02			0.02	0.01	

Timepoint Months from start of cobimetinib
Method Method used for sequencing - Whole Genome (WGS), Targeted pulldown or targeted amplicon
DNA Source Source of cells used for DNA extraction, CD19 purification of peripheral blood (PB) or Bone marrow (BM) or total BM.
Marrow Burden Product of marrow cellularity and tumor infiltrate

Supplementary Table 3. Primer sequences used for targeted amplicon sequencing

primer name	sequence (5'-3')	Tm	length	GC%	amplicon size (bp)	Amplicon GC%	Amplicon position (hg38)
KRAS_E2	gGCCTGCTGAAAATGACTGA	58.46	20	50	176	39.77	chr12:25245221-25245396
KRAS_E2	tgtatcaaagaatggtcctgcac	58.99	23	43.48			
KRAS_E3	tccagactgtgtttctccct	57.9	20	50	153	47.71	chr12:25227286-25227438
KRAS_E3	AAAGAAAGCCCTCCCCAGTC	59.59	20	55			
KRAS_E4	ACACAAAACAGGCTCAGGAC	58.32	20	50	225	31.1	chr12:25225463-25225687
KRAS_E4	gaagcaatgccctctcaaga	57.88	20	50			
BRAF_E15	tcataatgcttgctctgatagga	57.19	23	39.13	196	38.77	chr7:140753261-140753456
BRAF_E15	gcctcaattcttacCATCCACA	58.38	22	45.45			
MAPK_E2	TTGGAGGCCTTGCAGAAGAA	59.52	20	50	170	54.7	chr15:66435034+66435203
MAPK_E2	GAGACCTTGAACACCACACC	58.41	20	55			

PCR Stages	Number of Cycles
50ºC for 2 mins	1
70ºC for 20 mins	1
95ºC for 10 mins	1
95ºC for 15 s<br>60ºC for 30 s<br>72ºC for 1 min	10
95ºC for 15 s<br>80ºC for 30 s<br>60ºC for 30 s<br>72ºC for 1 min	2
95ºC for 15 s<br>60ºC for 30 s<br>72º C for 1 min	8
95ºC for 15 s<br>80ºC for 30 s<br>60ºC for 30 s<br>72ºC for 1 min	2
95ºC for 15 s<br>60ºC for 30 s<br>72ºC for 1 min	8
95ºC for 15 s<br>80ºC for 30 s<br>60ºC for 30 s<br>72ºC for 1 min	5
